# Identification, characterization, and expression analysis of WRKY transcription factors in *Gynostemma pentaphyllum* during tissue growth and cadmium stress

**DOI:** 10.3389/fpls.2025.1719872

**Published:** 2025-12-03

**Authors:** Yunyi Zhou, Lixiang Yao, Hai Lu, Zheng Xiong, Xinhao Li, Liying Yu, Chunliu Pan

**Affiliations:** 1 Guangxi Traditional Chinese medicine (TCM) Resources General Survey and Data Collection Key Laboratory, The Center for Phylogeny and Evolution of Medicinal Plants, National Center for TCM Inheritance and Innovation, Guangxi Botanical Garden of Medicinal Plants, Nanning, China; 2 National Engineering Research Center for Southwest Endangered Medicinal Materials Resources Development, Guangxi Botanical Garden of Medicinal Plants, Nanning, China

**Keywords:** Cd stress, *Gynostemma pentaphyllum*, genome-wide analysis, specific tissues, WRKY

## Abstract

*Gynostemma pentaphyllum*, a perennial medicinal herb, is widely distributed and exhibits remarkable adaptability to various environments. *WRKY* transcription factors play critical roles in regulating plant growth and development, as well as abiotic stress responses. However, the *WRKY* genes in *G. pentaphyllum* and their expression patterns under different tissues and Cd stress have not been thoroughly investigated, hindering our understanding of their role. In this study, 64 *WRKY* transcription factors identified in the *G. pentaphyllum* genome (*GpWRKY*) were phylogenetically grouped into three major groups and five subgroups and assigned a systematic nomenclature reflecting their positions across the 11 chromosomes. Comprehensive analyses of conserved domains, gene structures and motif features revealed a high degree of conservation with the gene family. Furthermore, seven pairs of segmental and tandem duplication events were detected, suggesting a contribution to the expansion of family. *Cis*-acting element analysis revealed the presence of hormone-related, stress-related, light-related, and development-related elements in the promoters of the majority of *GpWRKY* genes. Expression profiling analysis demonstrates that 64 *GpWRKY* genes were distinct in tissue-specific expression patterns and were substantially induced under Cd stress. Heterologous expression assay confirmed that *GpWRKY48*, one of the genes highly induced by Cd, positively regulates Cd tolerance. In this study, *GpWRKY* genes in *G. pentaphyllum* for the first time systematically identified and further investigated their physicochemical properties, evolution, and expression patterns, providing a theoretical basis for future studies on the functional characterization of *G. pentaphyllum WRKY* genes during plant growth and development, as well as Cd stress responses.

## Introduction

As one of the largest and most significant families of plant transcription factors, the WRKY family has been extensively reported to regulate growth, development, and stress responses (Wang et al., 2023a). With the genome-wide analyses of different species, the *WRKY* genes have been extensively identified in more species, such as 137 *WRKY* genes in *Panax ginseng*, 85 *WRKY* genes in *Neolamarckia cadamba*, 102 *WRKY* genes in poplar, 84 *WRKY* genes in *Gentiana macrophylla*, etc (Di et al., 2021; Gu et al., 2025; Ma et al., 2025; Xu et al., 2023). The WRKY protein contains a highly conserved 60 amino acids of WRKY domain, which has a WRKYGQK motif located in the N-terminal and a C2H2 or C2HC zinc finger motif in the C-terminus (Mahiwal et al., 2024; Xu et al., 2020). Furthermore, based on the number of WRKY structural domains and the type of zinc-finger motif, WRKY proteins are classified into three groups, which Group I possessing two WRKY domains with one C2H2 zinc finger motif, and Group II, and Group III possessing only one WRKY domain with a C2H2 or C2HC zinc finger motif (Chen et al., 2018, 2019). Group II can be further subdivided into IIa, IIb, IIc, IId, and IIe subgroups (Goyal et al., 2023). The WRKY proteins bind to the *cis*-acting DNA element W-box in the promoter region of target genes, positively or negatively regulating gene expression, and this interaction affects different developmental programs(growth and development, secondary metabolite, stress response) (Agarwal et al., 2011; Hsin et al., 2022).

Studies have suggested that *WRKY* gene serve as pivotal regulatory foundations in plant growth and development (Wang et al., 2023a). *AtWRKY1* and *AtWRKY75* were reported to be involved in leaf senescence in Arabidopsis, and *AtWRKY46*, *AtWRKY54* and *AtWRKY70* facilitates hypocotyl elongation through the brassinosteroid-mediated signaling pathway (Chen et al., 2017; Zhang et al., 2021, 2024). In strawberry, *FvWRKY50* was involved in regulating flowering time, leaf senescence, and fruit ripening (Chen et al., 2023b). *MaWRKY49* positively acted as a modulator of fruit ripening in *Musa acuminata* by activating the *MaPL3* and *MaPL11* expression (Liu et al., 2023). *GmWRKY46* was negatively regulated the proliferation, elongation and phosphorus absorption efficiency of hairy roots during phosphorus deficiency conditions in soybean (Liu et al., 2022). Furthermore, it has been reported that WRKY genes play an important regulatory roles in the biosynthesis of secondary metabolite, such as terpenoids, alkaloids, and phenolic compounds (Li et al., 2025). *LcWRKY17* positively promotes monoterpene synthesis by regulating the *LcTPS42* genes expression within *Litsea cubeba* (Gao et al., 2023). *CcWRKY7*, *CcWRKY29* and *CcWRKY32* activate the promoter of protoberberine biosynthetic gene *CcCNMT* to regulate protoberberine alkaloid biosynthesis in *Coptis chinensis*, respectively (Huang et al., 2023). In *Salvia miltiorrhiza*, *SmWRKY34* negatively regulates phenolic acid and tanshinone biosynthesis by targeting *SmRAS* and *SmGGPPS* genes, respectively (Shi et al., 2022).

Previous studies have demonstrated that *WRKY* gene play a crucial role in responses to both biotic and abiotic stress (Wang et al., 2023b). *SlWRKY51* functions as s positively regulatory in response to chilling stress by directly activating the expression of proline biosynthesis gene *SlP5CS1* (Wang et al., 2024). Under heat treatment condition, the overexpressed *MdWRKY75* has been shown to improve the degree of relative electrolyte leakage and contents of malondialdehyde(MDA) and proline, suggesting *MdWRKY75* responds to heat and positively regulates basal thermotolerance in *Malus domestica* (Zhang et al., 2024c). Increasing evidence suggests that the pivotal role of *WRKY* in cadmium(Cd), arsenic(As), aluminum(Al) and copper(Cu) metal stress responses (Huang et al., 2024b; Ma and Hu, 2024). Knockdown of *ZmWRKY64* disrupted Cd translocation in leaf and root cells, causing excessive Cd sequestration and decreasing Cd tolerance in maize (Gu et al., 2024). The As stress in rice rapidly induces not only the expression of *OsWRKY28*, a transcription factor associated with As transport, but also activates the MAPK cascade to phosphorylate *OsWRKY76* (Mirza et al., 2023; Wang et al., 2018). Under the same stress conditions, *OsWRKY71* may utilize GA signaling pathways to facilitate iron-mediated improvements in root system architecture (Mirza and Gupta, 2024). In soybean, *GmWRKY21* and *GmWRKY81* were induced by Al stress, and responsive to Al by regulating antioxidant enzyme genes (Han et al., 2022; Shu et al., 2022). Under conditions of Cu deficiency, *OsWRKY37* is involved in regulating flowering time and grain fertility by activating the expression of copper transporter gene *OsCOPT6* and yellow stripe-like gene *OsYSL16* in rice (Ji et al., 2024).

*Gynostemma pentaphyllum*(Thunb.) Makino, a well-known Chinese herbal plant, is widely used in the treatment of hyperlipidemia, hyperglycemia, and cancer. The main active components in *G. pentaphyllum* are saponins, polysaccharides, flavonoids and phytosterols, which act as anti-cancer and anti-atherogenic agents as well as affording neuroprotective and hepatoprotective properties (Su et al., 2021). The perennial creeping plant *G. pentaphyllum* possesses broad ecological adaptation and a wide suitable growth range, along with a high capacity for Cd uptake and tolerance (Li et al., 2023; Nookabkaew et al., 2016). This indicates that *G. pentaphyllum* plants possess specific adaptations for plant growth and Cd accumulation. Most studies have focused on the genes of terpenoids and saponins biosynthesis in *G. pentaphyllum* (Huang et al., 2024a; Qin et al., 2024; Zhang et al., 2024b), but few studies have focused on the genes of specific tissue growth and Cd response in *G. pentaphyllum* plants. Given the key roles of *WRKY* transcription factors in plant growth, development and stress response, 64 *GpWRKY* transcription factor genes were identified and characterized within the *G. pentaphyllum* genome. Subsequently, we conducted a comprehensive analysis of their structural features, phylogenetic relationships, conserved motifs, *cis*-regulatory elements, and synteny. Furthermore, the expression patterns of all 64 *GpWRKY* genes across different tissues and under Cd stress were assessed using RNA-seq data. Finally, the role of *GpWRKY4*8 in Cd tolerance was verified through heterologous expression in Arabidopsis. This study provides a valuable foundation for future research aimed at elucidating the functional roles of *GpWRKY* genes in mediating plant adapt to environmental ability.

## Experimental details

### GpWRKY proteins identification and sequence retrieval in *G. pentaphyllum*

Whole-genome sequence of *G. pentaphyllum*(PRJNA720501) was downloaded from NCBI (https://www.ncbi.nlm.nih.gov/). Arabidopsis WRKY sequences were acquired from the Arabidopsis Information Resource (TAIR) (https://www.arabidopsis.org/) Database. *G. pentaphyllum* sequences were aligned against Arabidopsis WRKY reference sequences using BLASTP(Parameters: e-value ≤ 1e^-5^). A Hidden Markov Model (HMM) was constructed from the Arabidopsis WRKY reference sequences by hmmsearch software (HMMER v3.0). Non-redundant sequences identified by BLASTP and HMMER were merged into a candidate *G. pentaphyllum* WRKY protein dataset. Candidate sequences were validated as *G. pentaphyllum* WRKY family members using pfam_scan.pl (v1.6) against the Pfam-A database (v33.1), retaining only those harboring the PF03106 WRKY domain.

### The physicochemical characterization of GpWRKY proteins

The presence and integrity of GpWRKY domains in candidate proteins were independently verified using the Conserved Domain Database (CDD, https://www.ncbi.nlm.nih.gov/Structure/cdd/wrpsb.cgi) and SMART (https://smart.embl-heidelberg.de/). Molecular weights and isoelectric points (pI) of GpWRKY proteins were calculated via the ExPASy ProtParam tool (https://web.expasy.org/protparam/). Subcellular localization predictions were generated using PSORT (https://www.genscript.com/psort.html) with default parameters.

### The phylogenetic analysis of GpWRKY proteins

Phylogenetic reconstruction of WRKY proteins was performed using validated sequences from *G. pentaphyllum* and Arabidopsis. Protein sequences were aligned with MAFFT (v7.427) using default parameters, followed by neighbor-joining tree construction in MEGA11 under the p-distance model with partial deletion treatment of missing data (site coverage cutoff: 50%). Branch confidence was assessed with 1,000 bootstrap replicates, and the final topology was annotated and visualized using iTOL v6 (https://itol.embl.de/).

### Identification of *cis*-acting regulatory elements, gene structure conserved motifs and gene duplications of *GpWRKY*

The *cis*-acting elements in the 2,000 bp promoter regions upstream of *GpWRKY* genes were predicted using PlantCARE(https://bioinformatics.psb.ugent.be/webtools/plantcare/html/). The protein motifs were analyzed via MEME Suite(https://meme-suite.org/meme/tools/meme) with default parameters. The Multiple Collinearity Scan toolbox (MCScanX) software (Parameters: e-value ≤ 1e^-5^) was employed to conduct analysis of gene duplication patterns in *G. pentaphyllum*. It includes identified segmental duplications and tandem duplications resulting from gene duplication events. Evolutionary divergence metrics(Ka: non-synonymous substitution rate; Ks: synonymous substitution rate) for duplicated gene pairs were calculated using KaKs Calculator 2.0. The TBtools was the platform used to analyze the data.

### Analysis of *GpWRKY* expression profiles based on transcriptomics analysis

*G. pentaphyllum* specimens were obtained from the Guangxi Botanical Garden of Medicinal Plants(Nanning, China) and cultivated in a greenhouse under 16-h light/25°C day and 8-h dark/20°C night cycles. The fifteen samples from the five tissues(root, stem, leaf, flower and fruit) were taken for transcriptomics analysis. The experimental methods and analytical approaches for transcriptomics (root, stem, leaf, flower and fruit) were adapted from previous transcriptomics (Zhou et al., 2023).

For Cd stress, the seedlings culture method and treatment were taken as described by previous studies (Li et al., 2022, 2023), with the treatment solution concentrations of 0, 25 and 100 μM CdCl_2_. Fresh leaf samples of nine samples from the three conditions were acquired for further analysis. Transcript profile of Cd-responsive *GpWRKY* genes was carried out from an earlier transcriptome dataset (Zhou et al., 2023).

The differential expressed genes(DEGs) were calculated by using the NOISeq method, employing a |log2 (fold change) |>2 and p-value <0.05. The *GpWRKY* gene expression values were calculated by log2(FPKM) and normalized via Z-score normalization. The heatmap were drawn using R version 4.1.0 by gene expression values of means.

### Quantitative real-time PCR analysis

Total RNA from *G. pentaphyllum* tissues under Cd exposure was isolated using the Eastep™ Super Total RNA Extraction Kit(Promega). cDNA synthesis employed 1 μg RNA with HiScript III RT SuperMix(+gDNA wiper; Vazyme). Sixteen *GpWRKY* genes were analyzed by qRT-PCR using LightCycler 96(Roche) with gene-specific primers (designed via Primer 5.0) (**Supplementary Table S5**). Actin served as the reference gene. Relative expression was calculated using the 2**^–ΔΔCt^** method, revealing concordant patterns between qRT-PCR and transcriptomic data.

### Overexpression of the *GpWRKY48* gene in Arabidopsis

The CDS sequence of *GpWRKY48* without the stop codon was inserted into the plasmid vector pCAMBIA1301, which was transformed into *Agrobacterium tumefaciens* strain GV3101 to infiltrate inflorescence using the Agrobacterium-mediated floral dipping method in Arabidopsis. The true transformed T0 seeds were selected by kanamycin, and stable *GpWRKY4*8 overexpressed lines were obtained at T3 generation. We used the *GpWRKY4*8-OE(*GpWRKY48* overexpressed) lines to record initial growth phenotypes. Wild-type Arabidopsis(Columbia ecotype) plants were used for phenotypic comparison with the transgenic lines. 7-day-old seedlings were grown on 1/2 MS media with or without 50μM CdCl_2_ for one week and accessed the differences in root growth. 30-day-old seedlings grown in peat soil were subjected to Cd treatment. Each pot was irrigated with 30 mL of 200 μM CdCl_2_ solution every three days, and phenotypic differences were assessed after 10 days. The enzyme activities of superoxide dismutase(SOD), peroxidase(POD), catalase(CAT) and the contents of malondialdehyde(MDA) were assessed and analyzed utilizing the methods as previously described (Pan et al., 2023).

### Statistical analysis

The SPSS v 26.0 software was used to analyze the statistical data. All values were expressed as the means ± standard deviations. The one-way ANOVA with Duncan’s multiple-range test was used to test the data. The p < 0.05 was considered to be statistically significant. The graphs were drawn using GraphPad Prism 8.

## Results

### Identification of sixty-four *WRKY* genes in *G. pentaphyllum*

**Figure 1:**
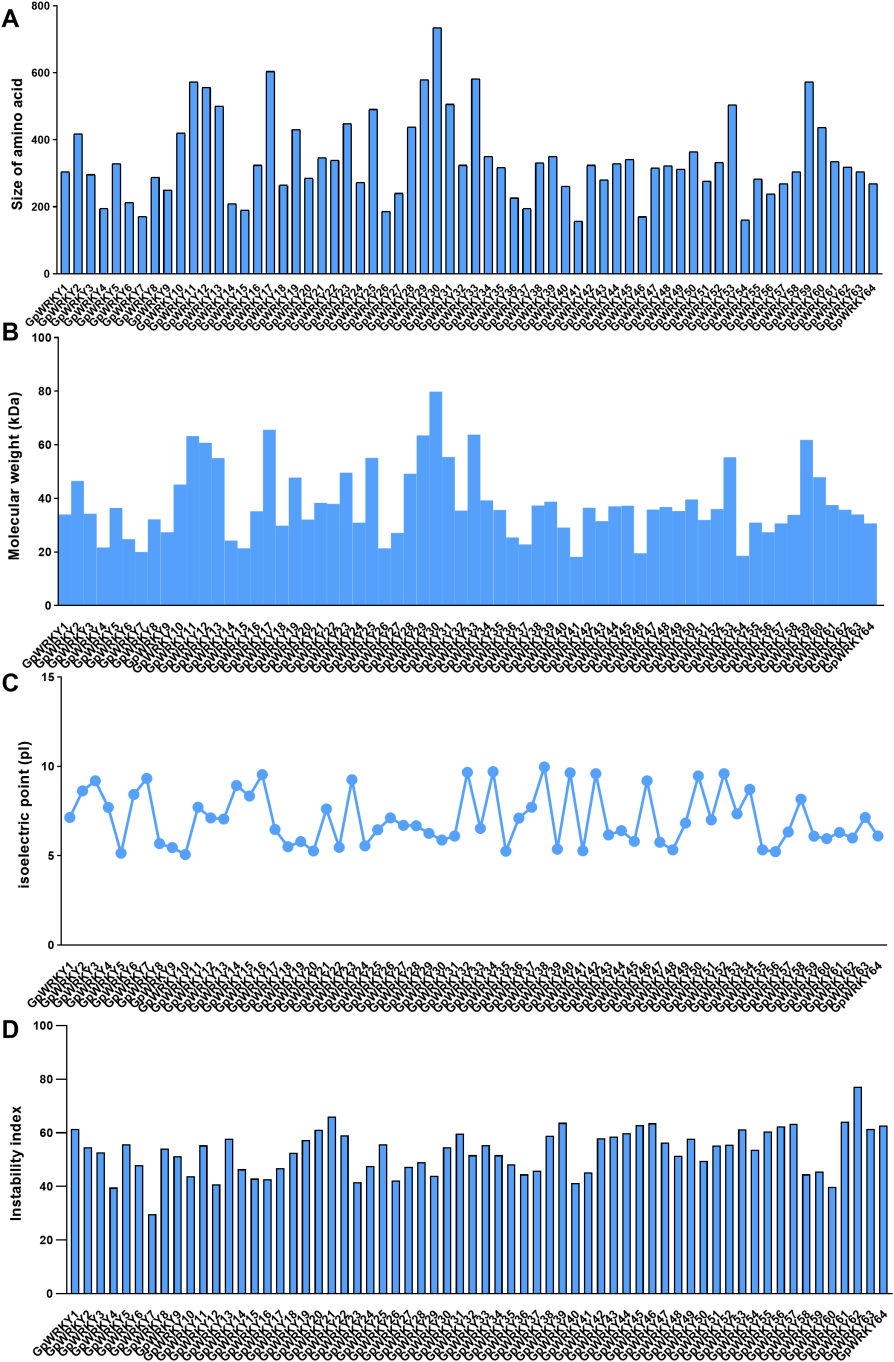
The physiochemical analysis of the predicted GpWRKY proteins: **(A)** The size of amino acids. **(B)** The molecular weight. **(C)** The isoelectric point. **(D)** The instability index.

A total of 64 *GpWRKY* genes were obtained from *G. pentaphyllum* genomic and transcriptomic data and assigned new names from *GpWRKY1* to *GpWRKY64* according to their gene structure and gene ID (**Supplementary Table S1**). The physicochemical properties of GpWRYK proteins were successfully analyzed (**Figure 1**; **Supplementary Table S1**). The size of amino acids ranged from 157 to 735, with predicted molecular weights ranging from 18.19 to 79.85 kDa (**Figure 1A**). GpWRKY41 had the largest molecular weight of 79.85 kDa and was composed of 735 amino acids, whereas GpWRKY30 had the smallest molecular weight of 18.20 kDa and contained 157 amino acids (**Figure 1B**). The isoelectric points of GpWRKY proteins ranged from 5.07(GpWRKY10) to 9.97(GpWRKY38) (**Figure 1C**). The instability index ranges from 29.61(GpWRKY7) to 77.21 (GpWRKY62) (**Figure 1D**). Predictive subcellular localization analysis showed that 61 GpWRKY proteins were localized in the nucleus, whereas GpWRKY28, GpWRKY38 and GpWRKY49 proteins were localized in the peroxisome, plasma membrane and cytoskeleton, respectively (**Supplementary Table S2**).

### Phylogenetic analysis and classification of GpWRKY proteins in *G. pentaphyllum*

**Figure 2:**
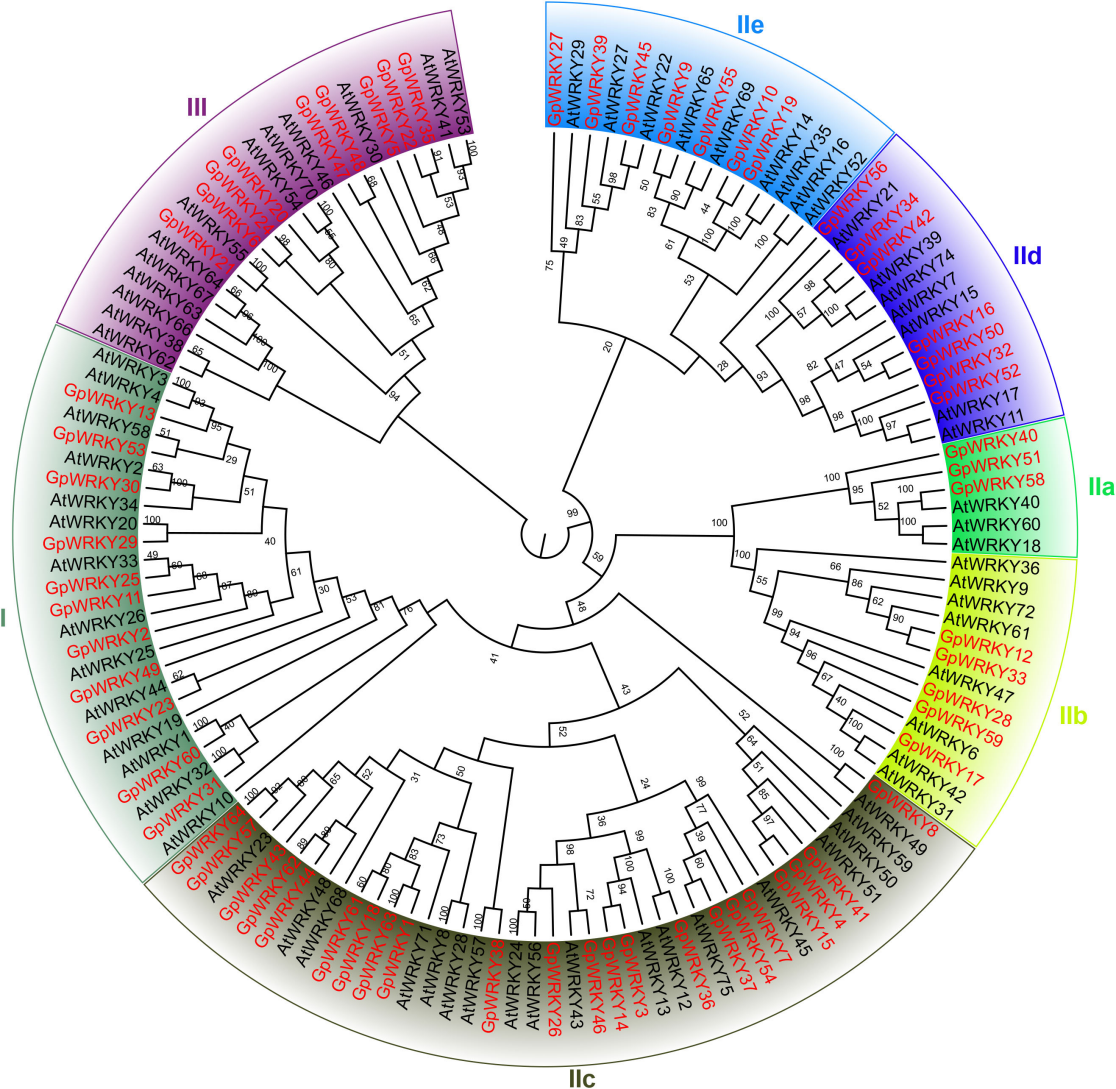
Phylogenetic analysis of the relationships between the WRKY proteins of *G. pentaphyllum* and *A. thaliana*. The I, IIa, IIb, IIc, IId, IIe, and III represent the different subfamilies.

**Figure 3:**
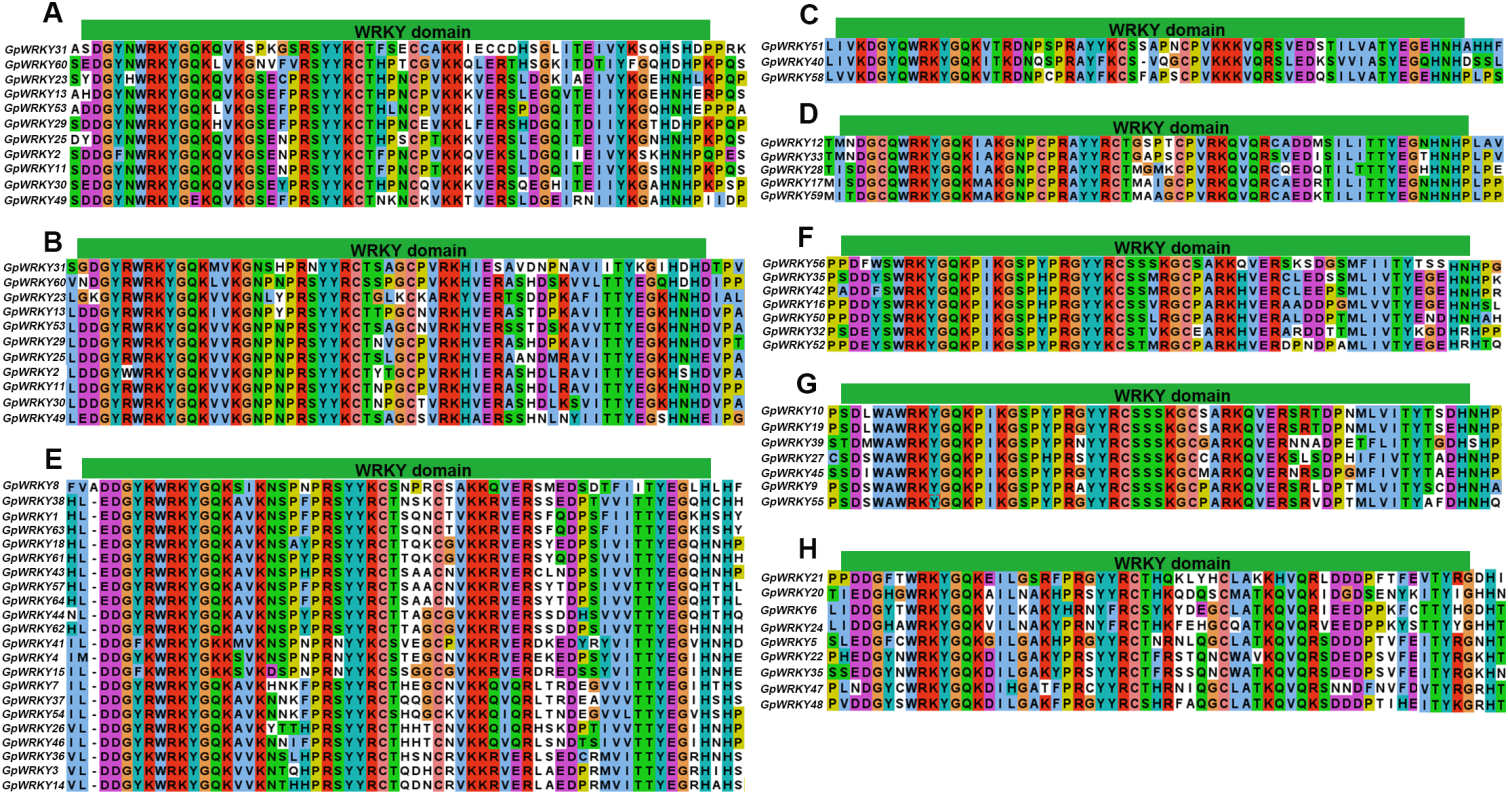
Conserved WRKY domain of seven subfamilies in the GpWRKY proteins. **(A)** N-terminal WRKY domains of Family (I) **(B)** C-terminal WRKY domains of Family (I) **(C)** N-terminal WRKY domains of Family IIa. **(D)** N-terminal WRKY domains of Family IIb. **(E)** N-terminal WRKY domains of Family IIc. **(F)** N-terminal WRKY domains of Family IId. **(G)** N-terminal WRKY domains of Family IIe. **(H)** N-terminal WRKY domains of Family III.

To gain insight into the subfamily classification of the WRKY family members of *G. pentaphyllum*, a phylogenetic tree was constructed using the neighbor-joining analysis with 64 GpWRKY and 72 AtWRKY proteins (**Figure 2**). The 64 GpWRKY proteins were categorized into subfamilies: family I, IIa, IIb, IIc, IId, IIe, and III. Among then, family IIc had 22 GpWRKY members, followed by families I(11), III(9), IId(7), IIe(7), IIb(5) and IIa(3).The WRKY structural domain contained 60 amino acids was selected for analysis subfamilies. Family I contained two WRKY structural domains in the N-terminal and C-terminal of amino acid sequence, while subfamilies IIa, IIb, IIc, IId, IIe, and III contained one domain. A total of 60 GpWRKY proteins were identified to contain the highly conserved WRKYGQK conserved sequence (**Figure 3**), whereas others varied by individual amino acids(GpWRKY49 proteins had WRKYGEK conserved sequence, and GpWRKY41, GpWRKY4, and GpWRKY15 had WRKYGKK conserved sequence). Interestingly, most of GpWRKY proteins in subfamily IIc have one amino acid missing in WRKY structural domain, which needs to be investigated further.

### Identification of the gene structure, conserved motifs, and *cis*-acting elements of *GpWRKY* genes in *G. pentaphyllum*

**Figure 4:**
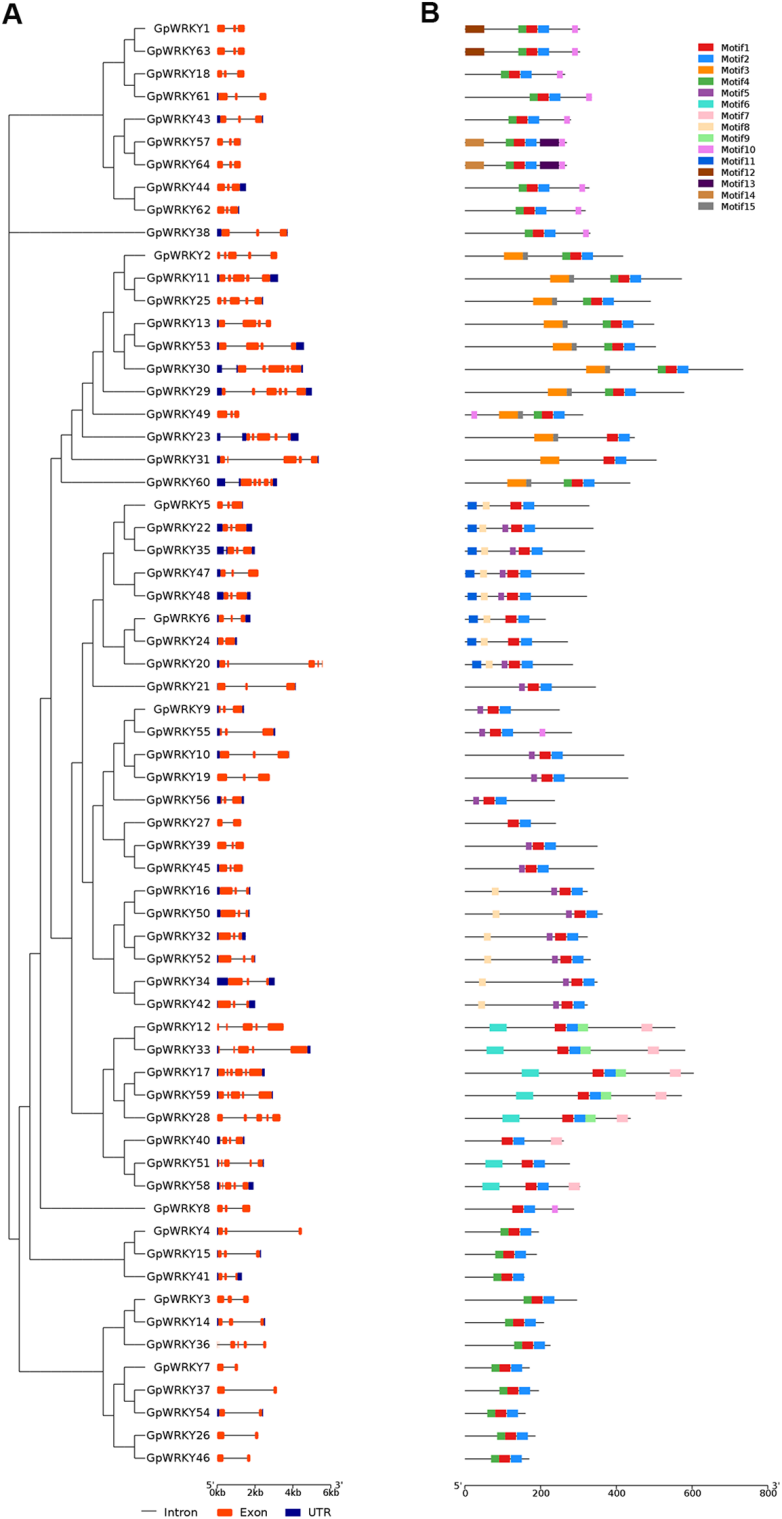
Gene structure and conserved motif analysis of *GpWRKY* genes. **(A)** Gene structure. **(B)** Motif composition. The details of motif sequence were illustrated in **Supplementary Table S3**.

To understand the untranslated region(UTR), coding sequence(CDS) and introns of *GpWRKY* genes, a structural map was constructed based on the *G. pentaphyllum* genome sequence (**Figure 4A**; **Supplementary Table S3**). The structural composition of *GpWRKY* genes revealed that 46 *GpWRKY* genes contained UTRs. 64 *GpWRKY* genes contained introns, of which 37(57.8%) contained two introns, 14(21.8%) contained four, 7(10.9%) contained one, and 3(4.6%) contained three or five. The distribution of 15 conserved motifs identified in *64 GpWRKY* genes were analyzed (**Figure 4B**). All of *GpWRKY* genes had motif 1 and 2, and motif 1 is the WRKY structural domain. Motif 3, 10 and 15 were unique to family I. Motif 9 was unique to family IIb. Motif 12,13 and 14 were unique to family IIc. Motif 11 was unique to family III. Motif 4 was shared in family I and IIc. Motif 6 and 7 was shared in family IIa and IIb. Motif 5 was shared in family IId, IIe and III. Motif 8 was shared in family IId and III.

**Figure 5:**
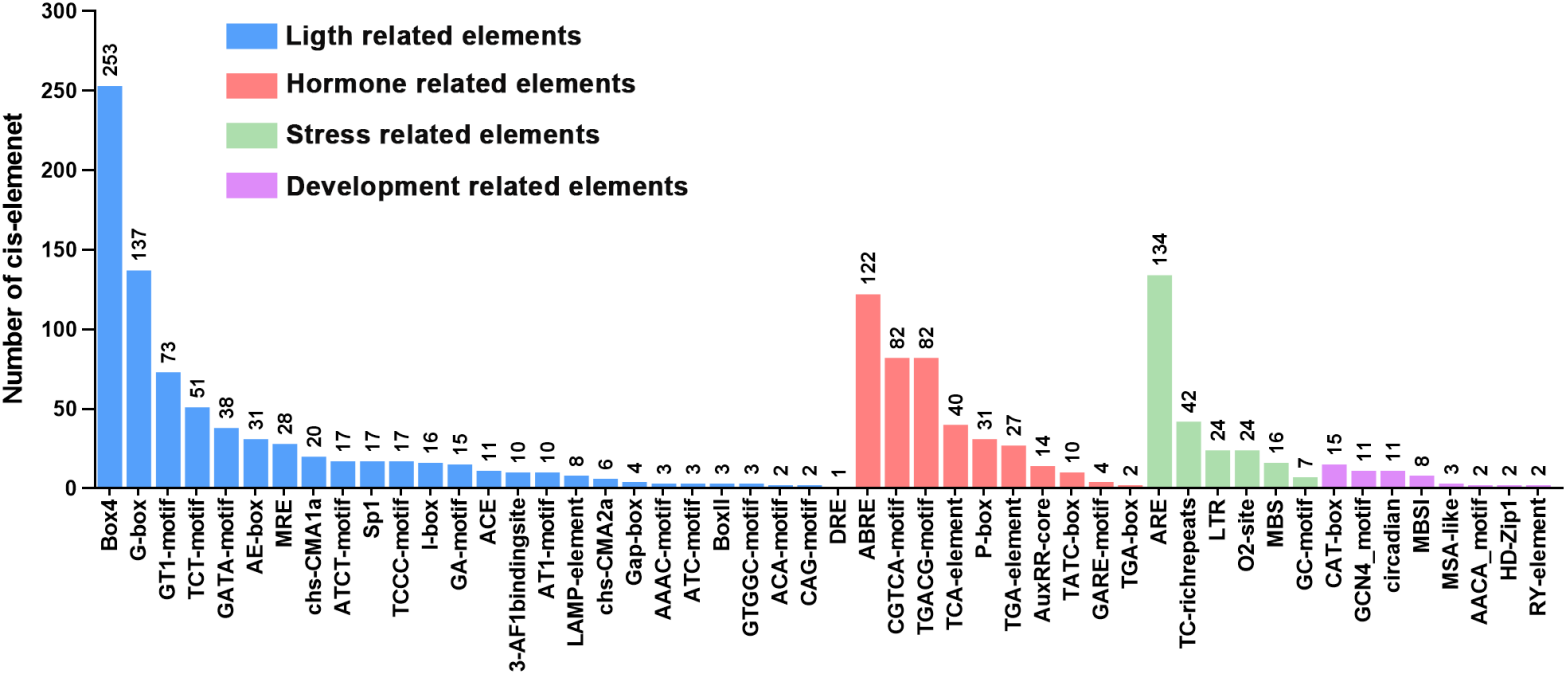
*Cis*-acting elements in the promoters of *GpWRKY* genes.

To elucidate the potential functions of the *GpWRKY* family members in plant growth and development, response to plant hormones, and environmental stresses, the *cis*-acting elements in the upstream promoter regions of the *GpWRKY* genes were analyzed (**Figure 5**). A total of 50 *cis*-elements in *GpWRKY* genes were identified and categorized into four categories(hormone-related, stress-related, light-related, and development-related elements). The light-related elements were found to have the highest number of *cis*-acting element types, including Box4, G-box, GT1-motif, TCT-motif, ATA-motif and AE-motif and so on. Hormone-related elements had 10 types of *cis*-acting elements, including abscisic acid responsiveness(ABRE), MeJA responsiveness(CGTCA-motif and TGACG-motif), salicylic acid responsiveness(TCA-element), auxin responsiveness(AuxRR-core, TGA-element, and TGA-box), gibberellin-responsive elements(GARE motif, P-box, and TATC-box). Six stress-related *cis*-elements were also detected, including anaerobic induction elements(ARE), defense and stress responsive elements (TC-rich repeats), low-temperature responsive elements(LTR), *cis*-acting regulatory elements involved in zein metabolism regulation(O2-site), drought stress-responsive elements(MBS), anoxic-specific inducibility(GC motif). Eight development-related *cis*-elements were identified, such as meristem expression elements(CAT-box), seed specific regulation elements(RY-element), flavonoid biosynthetic genes regulation elements(MBSI), and endosperm expression elements(GCN4_motif). Specifically, 253 Box4, 137 G-box and 122 ABRE elements were distributed in promoter regions of 64 *GpWRKY* genes.

### Chromosome distribution and synteny analysis of *GpWRKY* genes in *G. pentaphyllum*

**Figure 6:**
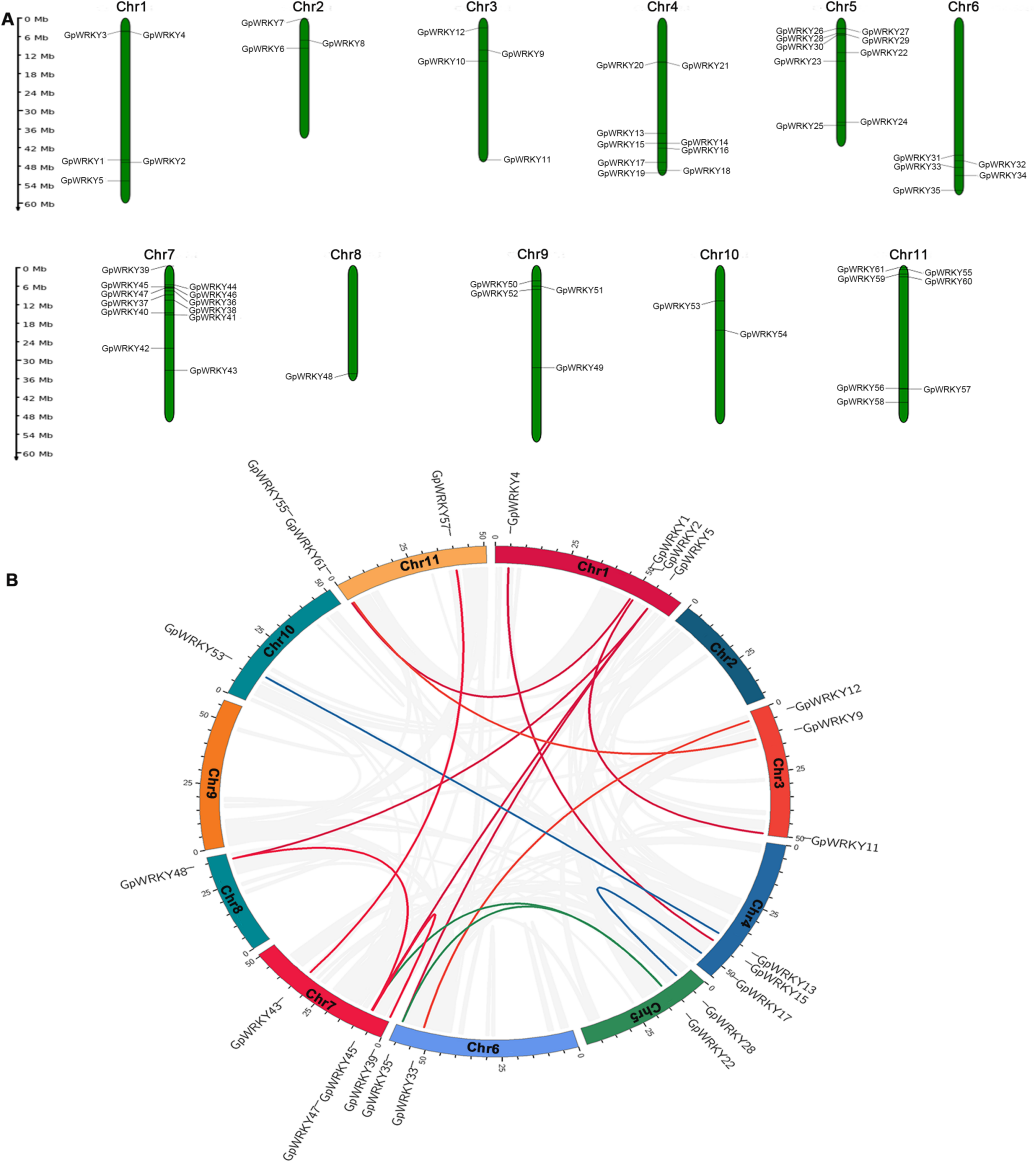
Chromosome distribution and gene duplication relationship of *GpWRKY* genes. **(A)** Chromosome location. **(B)** Gene duplication relationship.

To determine the distribution of the *GpWRKY* genes in *G. pentaphyllum* genome, the chromosomal mapping of 64 *GpWRKY* genes was conducted and visualized (**Figure 6A**). The results showed that 61 *GpWRKY* genes were unevenly distributed across the 11 chromosomes, while 3 *GpWRKY* genes(*GpWRKY62*, *GpWRKY63* and *GpWRKY64*) remained unlocalized and distributed in the unmounted fragments(JAHXMR010000013.1, JAHXMR010000086.1 and JAHXMR010000462.1). Among them, the majority of *GpWRKY* genes were located on Chr 7(*GpWRKY36*-*GpWRKY47*,18.7%), Chr 4(*GpWRKY13*-*GpWRKY21*,14.0%), Chr 5(*GpWRKY22*-*GpWRKY30*,14.0%) and Chr 11(*GpWRKY55*-*GpWRKY61*,10.9%).

To investigate the segmental and tandem duplication events among *GpWRKY* genes in *G. pentaphyllum* genome, a collinearity analysis was conducted (**Figure 6B**). The results revealed that 16 duplication events occurred in the *GpWRKY* gene family across with 9 chromosomes except Chr 2 and Chr 9. The chromosome 1 showed the largest number of duplication events, and had 6 pairs of segmental duplication events(*GpWRKY4*/*GpWRKY15*, *GpWRKY1*/*GpWRKY61*, *GpWRKY2*/*GpWRKY11*, *GpWRKY5*/*GpWRKY35*, *GpWRKY5*/G*pWRKY47*, *GpWRKY5*/*GpWRKY48*). The chromosome 7 showed 1 pair of tandem duplication event (*GpWRKY39*/*GpWRKY45*). The Ka, Ks, and Ka/Ks values for the *GpWRKY* gene family were identified to characterize the gene duplication events. 2016 gene pairs were examined and assessed the evolutionary selection pressure of *GpWRKY* genes (**Supplementary Table S4**). The results showed that the Ks values between 2016 *GpWRKY* gene pairs ranged from 0.02 to 4.47. The 2016 *GpWRKY* gene pairs had Ka/Ks values < 1, suggesting that these genes had a strong purifying selection during evolution.

### Identification of key *GpWRKY* genes expression among different tissues and Cd stress

**Figure 7:**
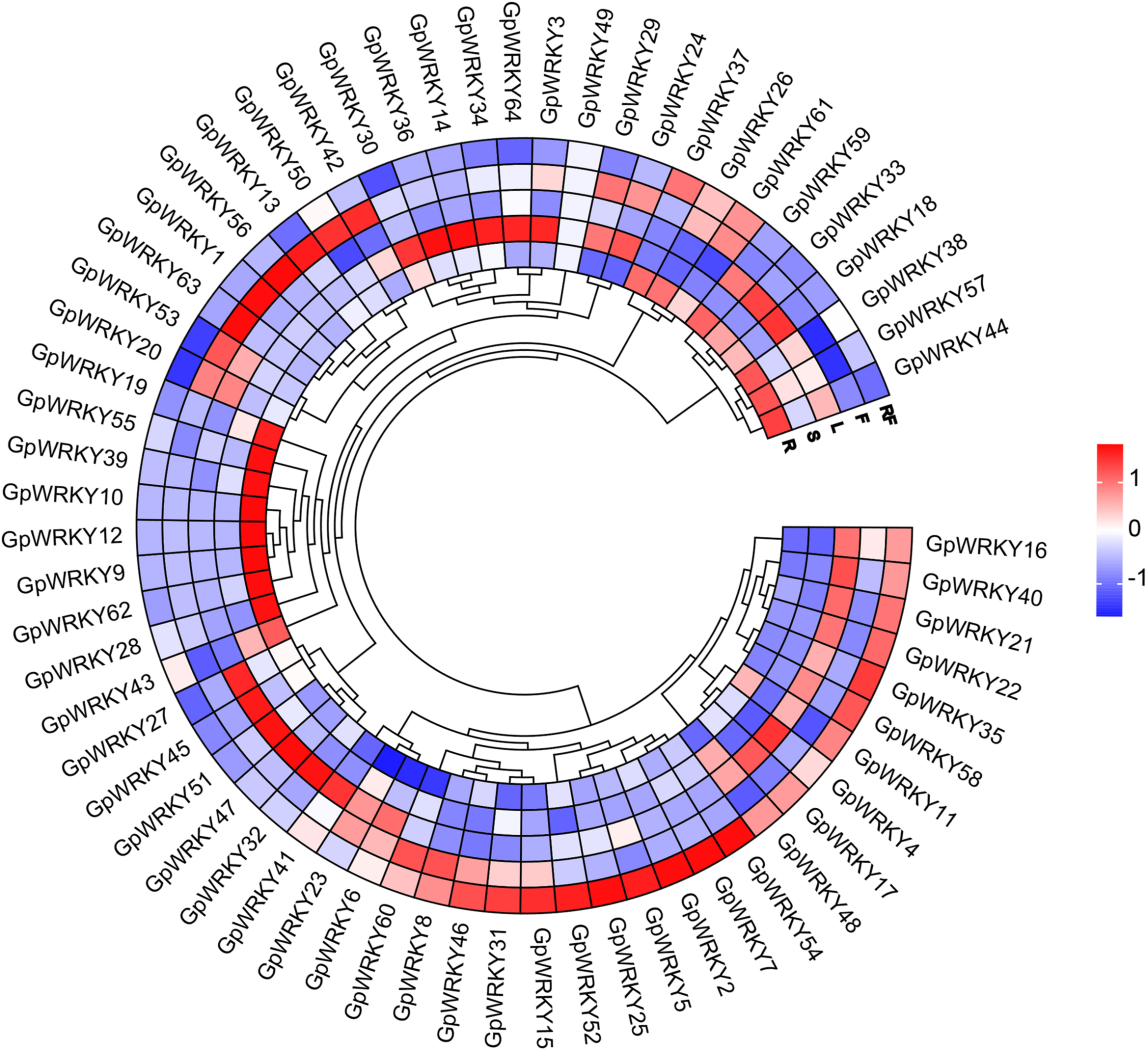
Expression patterns of 64 *GpWRKY* genes in *G. pentaphyllum* five tissues(R: roots, S: stems, L: leaves, F: flowers, RF: ripe fruits). Red, white, and blue indicate high, medium and low levels of expression, respectively.

To investigate the expression patterns of *GpWRKY* genes in different tissues of *G. pentaphyllum*, the expression data of 64 *GpWRKY* genes were taken from the transcriptomes of roots, stems, leaves, flowers and fruits. In this study, the FPKM value of each *GpWRKY* gene was used to calculated its expression level in different tissues. The results indicated that the expression of most *GpWRKY* genes were higher in leaves and fruits than in other tissues (**Figure 7**). 64 *GpWRKY* genes were expressed in at least two tissues, whereas *GpWRKY49* showed low expression across five tissues. Several genes exhibited tissue-specific expression patterns: *GpWRKY2*8, *GpWRKY62*, *GpWRKY9*, *GpWRKY12*, *GpWRKY10*, *GpWRKY39*, *GpWRKY55* and *GpWRKY19* gene were high expressed in roots. *GpWRKY30*, *GpWRKY36*, *GpWRKY14*, *GpWRKY34*, *GpWRKY64* and *GpWRKY*3 genes were high expressed in stems. *GpWRKY41*, *GpWRKY32*, *GpWRKY47*, *GpWRKY51*, *GpWRKY45* and *GpWRKY27* genes were high expressed in leaves. *GpWRKY63*, *GpWRKY1*, *GpWRKY56*, *GpWRKY13*, *GpWRKY50* and *GpWRKY42* genes were high expressed in flowers. *GpWRKY54*, *GpWRKY7*, *GpWRKY2*, *GpWRKY5*, *GpWRKY25* and *GpWRKY52* genes were high expressed in fruits. Notably, their expression in other organs was not significant. These results suggest that these *GpWRKY* genes are high expressed in specific tissues and that these genes may play important role in the tissue growth and development of *G. pentaphyllum*.

**Figure 8:**
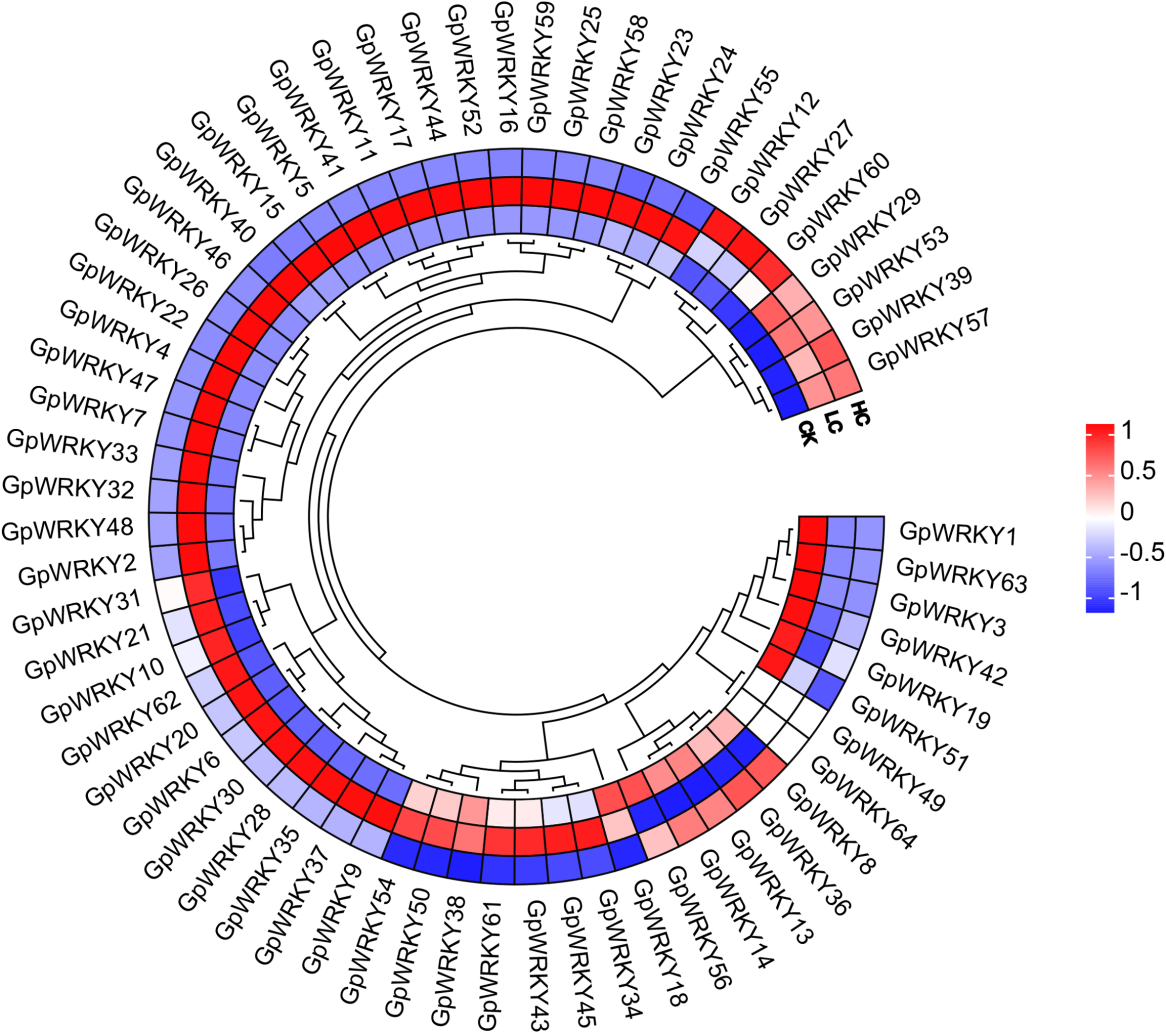
Expression patterns of 64 *GpWRKY* genes in *G. pentaphyllum* seedlings across control(CK), low-concentration(25 μM CdCl_2_, LC), and high-concentration(100 μM CdCl_2_, HC) Cd treatments. Red, white, and blue indicate high, medium and low levels of expression, respectively.

To investigate the expression patterns of *GpWRKY* genes in Cd stress, the *G. pentaphyllum* seedlings under the control group(CK), and different Cd treatments(Cd25, LC and Cd100, HC) were determined to analysis. Compared to the CK group, a total of 50 *GpWRKY* genes exhibited high expression under either LC or HC treatment (**Figure 8**). Among these, 7 genes(*GpWRKY57*, *GpWRKY39*, *GpWRKY53*, *GpWRKY29*, *GpWRKY60*, *GpWRKY27* and *GpWRKY12*) were substantially high expressed under Cd treatment. Conversely, the expression levels of *GpWRKY1*, *GpWRKY63*, *GpWRKY3*, *GpWRKY42*, *GpWRKY19* and *GpWRKY5*1 were low expressed in response to Cd stress. This significant differential expression of 13 *GpWRKY* genes under Cd stress implied their potential involvement in the response of *G. pentaphyllum* to Cd stress.

**Figure 9:**
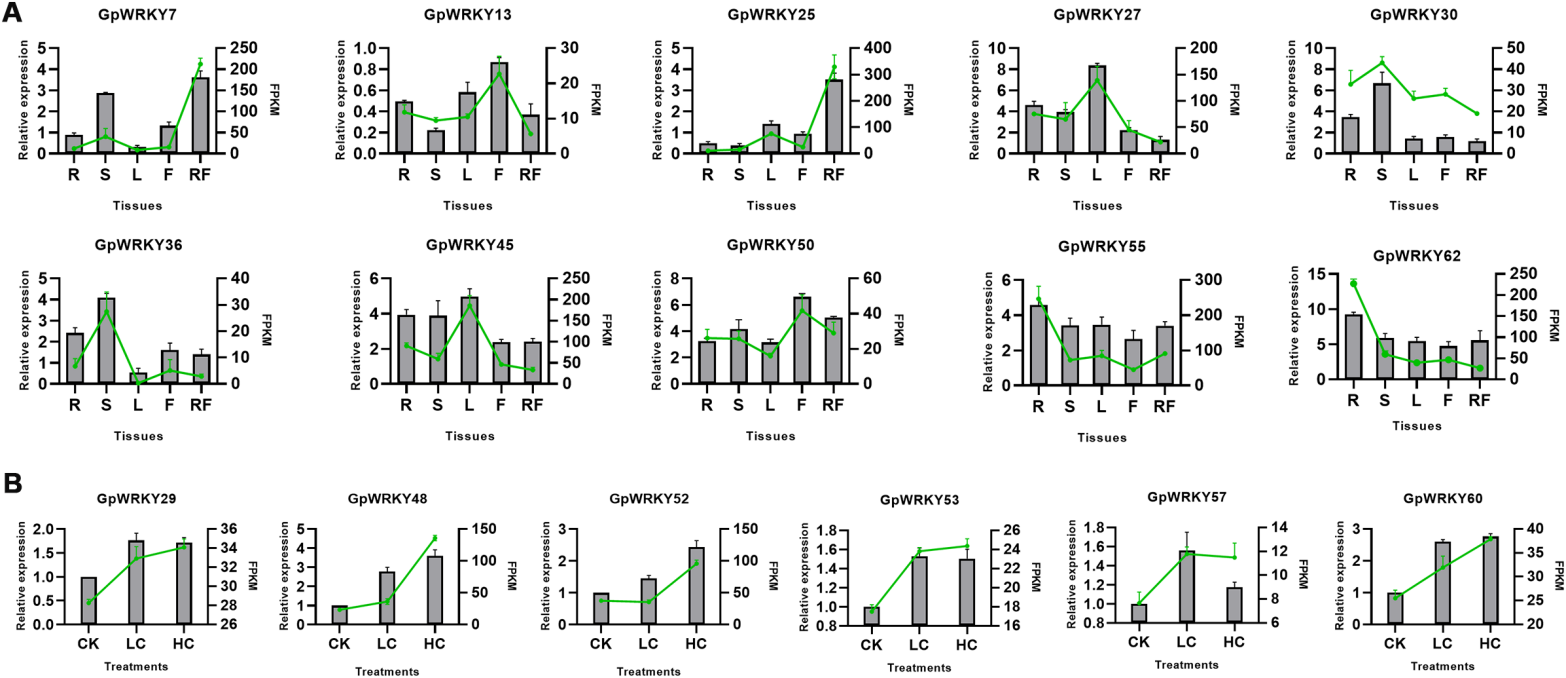
Analysis of *GpWRKY* gene expression by qRT-PCR and RNA-seq. **(A)** Expression patterns of 10 *GpWRKY* genes in five tissues (R: roots, S: stems, L: leaves, F: flowers, RF: ripe fruits). **(B)** Expression patterns of 6 *GpWRKY* genes under Cd treatment (CK: the control group; LC: 25 μM CdCl_2_ treatment group; HC: 100 μM CdCl_2_ treatment group). qRT-PCR data (gray columns) are presented as means ± SD(n = 3). RNA-seq data(green lines) are presented as mean ± SD(n = 3).

To confirm the transcript analysis, the qRT-PCR profile of 10 *GpWRKY* genes in five tissues and 6 *GpWRKY* genes in Cd treatment were examined (**Figure 9**), respectively, which showed that the gene expression trend was consistent with the transcriptome data, indicating that the transcriptome data analysis results were reliable.

### Overexpression of *GpWRKY48* increased Cd resistance in *G. pentaphyllum*

**Figure 10:**
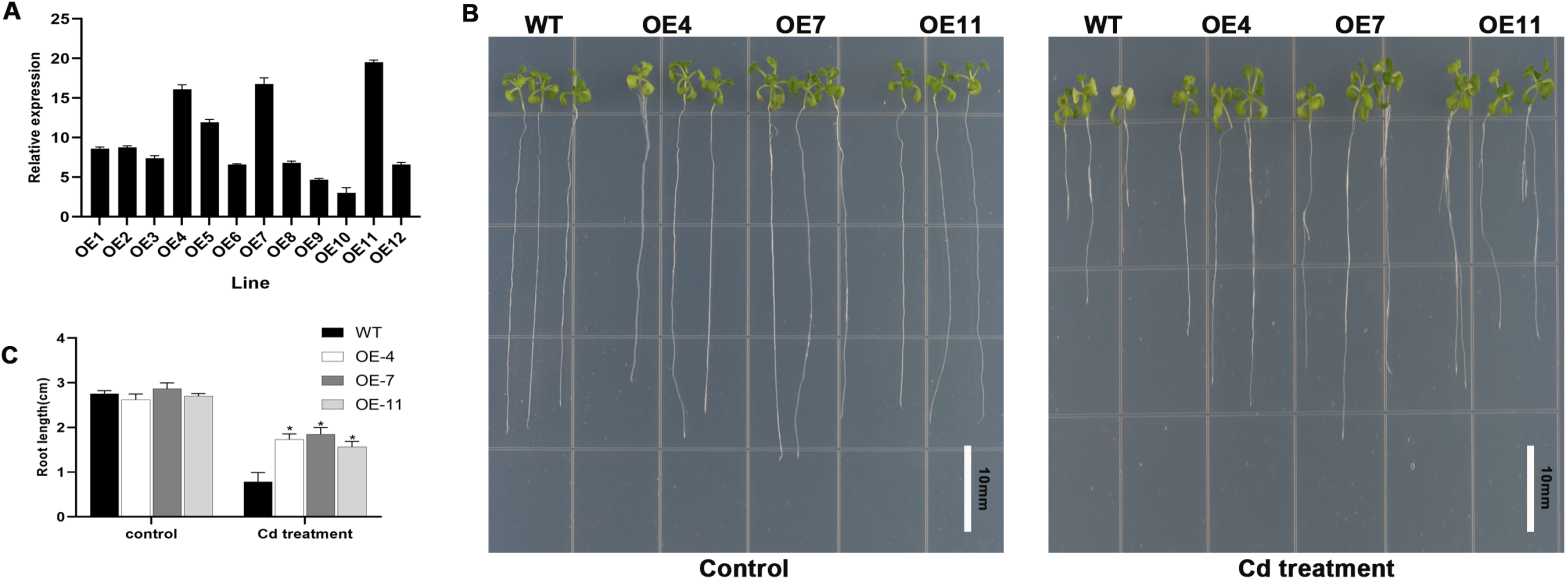
Analysis of overexpression of *GpWRKY48* line in response to Cd stress in Arabidopsis. **(A)** GpWRKY48 expression in transgenic lines. **(B)** Phenotype of control plants (WT) and overexpression plants (OE-4, OE-7 and OE-11) before and after Cd treatment on a 1/2 MS medium. **(C)** Root length of control plants (WT) and overexpression plants (OE-4, OE-7 and OE-11) before and after Cd treatment on a 1/2 MS medium. The bars represent the mean ± SD of three biological repeats. Asterisks indicate statistically significant differences from WT(*P < 0.05).

**Figure 11:**
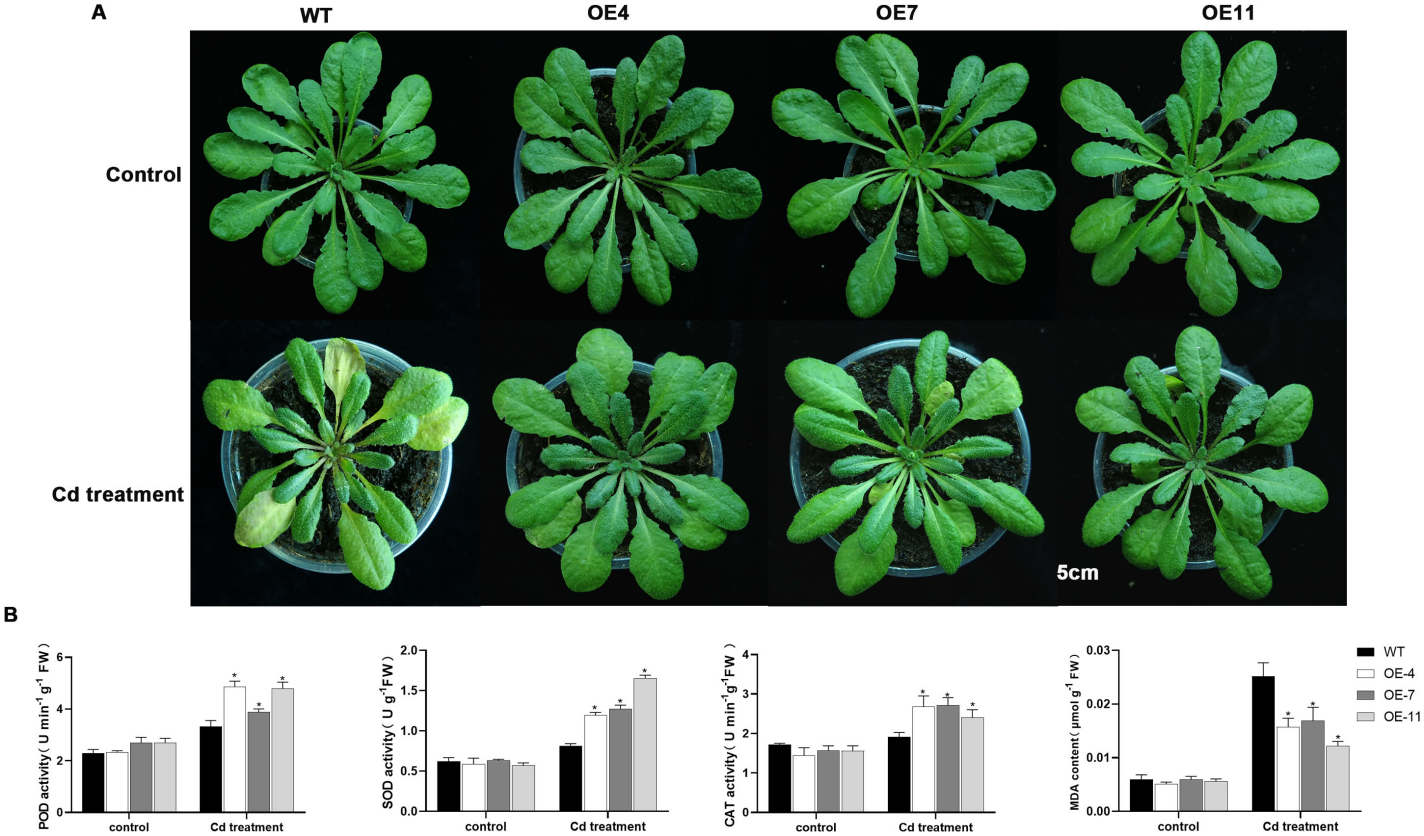
Overexpression of *GpWRKY48* enhances Cd tolerance in Arabidopsis. **(A)** Phenotype of control plants (WT), overexpression plants(OE-4, OE-7 and OE-11) before and after Cd treatment. **(B)** POD, SOD, CAT activity and MDA content in the wild-type and transgenic lines after Cd treatment for 7 days. The bars represent the mean ± SD of three biological repeats. Asterisks indicate statistically significant differences from WT(*P < 0.05).

*GpWRKY48* was found to be one of the genes that was most remarkably induced by Cd stress, suggesting that it possibly plays a significant role in Cd resistance. To confirm this assumption, the agrobacterium-mediated floral-dip method was employed to *GpWRKY48* in Arabidopsis. qRT-PCR analysis showed that the heterologous expression of *GpWRKY48* was markedly high in Arabidopsis, and the transcript levels in OE-4, OE-7 and OE-11 lines were higher than in the other lines (**Figure 10A**). Therefore, we selected the homozygous lines OE-4, OE-7 and OE-11 in T3 plants for further research. After 7 days of Cd exposure, the root lengths of OE-4, OE-7 and OE-11 plants were 121.3%, 136.2%, and 100.1% higher than that of the wild-type plants, respectively (**Figures 10B, C**). When subjected to Cd treated or untreated conditions, no significant phenotypic differences were observed between wild-type Arabidopsis and overexpression lines, except for a more pronounced leaf yellowing in the wild-type plants under Cd treatment conditions (**Figure 11A**). Furthermore, assessment of several physiological parameters revealed significant differences between the wild-type plant and overexpression lines. The activities of SOD, POD and CAT in transgenic lines were dramatically higher than in wild-type plants, while the content of MDA in transgenic lines were significantly lower than in wild-type plants (**Figure 11B**). We concluded that *GpWRKY48* enhanced Cd tolerance in Arabidopsis, suggesting that it functions as a positive regulator of Cd tolerance in *G. pentaphyllum*.

## Discussion

*G. pentaphyllum* is a valuable medicinal plant whose leaves contain several saponins, polysaccharides, flavonoids, phytosterols and other bioactive ingredients that are often used in health care and disease treatment (Su et al., 2021). *G. pentaphyllum* also exhibits broad ecological adaptation and a suitable growth range, along with a high capacity for Cd absorption and tolerance (Li et al., 2022; Nookabkaew et al., 2016). The *WRKY* transcription factor family plays crucial roles in plant growth, development and stress regulation. Genome-wide identification of *WRKY* TFs has been conducted in several medicinal plants, including *N. cadamba* (Xu et al., 2023), *Sophora flavescensrice* (Li et al., 2024) and *Gentiana macrophylla* (Gu et al., 2025). Recent studies on *G. pentaphyllum* have primarily focused on the analysis of bioactive components and their related regulatory mechanisms (Huang et al., 2024a; Liang et al., 2025; Qin et al., 2024), however, the regulatory roles of *WRKY* TFs in the *G. pentaphyllum* growth and development, especially under stress conditions, remain largely unknown. In this study, 64 *GpWRK*Y members were identified and renamed *GpWRKY*1-*GpWRKY64*.

Based on the phylogenetics and sequence analyses, the 64 *GpWRKY* genes are classified into three main groups, with the second group containing the highest number of members and further subdivided into five subgroups (**Figure 2**), consistent with the classification scheme established in Arabidopsis plants (Wu et al., 2005). Notably, the II group has the highest number of *GpWRKY* genes and contains 6 gene duplications(*GpWRKY4*, *GpWRKY15*, *GpWRKY1*, *GpWRKY61*, *GpWRKY39* and *GpWRKY45*), indicating potential gene duplication during the II group evolutionary history of this group. Previous studies suggest that the tandem and segmental duplication events in the expansion of the *WRKY* gene family were mainly due to segmental duplication events and divergent selection (Yin et al., 2013). In this study, 7 pair of segmental and tandem duplication events have contributed to the expansion of *GpWRKY* gene family (**Figure 6B**). The calculated Ka/Ks ratios calculations of *GpWRKY* gene pairs were all less than one (**Supplementary Table S4**). Our findings are consistent with findings from the *SfWRKY* gene family in *S. flavescens* (Li et al., 2024). The duplicated *GpWRKY* genes may have undergone negative selection, implying that functional constrainsts and potential roles in diverse regulatory processes during *G. pentaphyllum* growth and development.

The protein sequence alignment found that group IIc and group I had sequence variation in the WRKY structural domain(GpWRKY49, GpWRKY41, GpWRKY4, and GpWRKY15). Through multiple sequence alignment, it was found that the WRKYGQK motif in WRKY49 had mutated to WKKYGEK, while in GpWRKY41, GpWRKY4, and GpWRKY15, it had mutated to WRKYGKK (**Figure 3**). Previous studies suggested that such variations originate from evolutionary variations of ancestral WRKY transcription factors in rice and Arabidopsis over an extended period (Wu et al., 2005). It has been demonstrated that the WRKY domain variations may impact the interaction function between the WRKY gene and their target genes (Ciolkowski et al., 2008). For instance, soybean GmWRKY6 and GmWRKY21 with an altered WRKYGKK sequence in their N-terminal, lost the ability to bind to the W-box(TTGAC) (Zhou et al., 2008). In contrast, tobacco NtWRKY12 with WRKYGKK sequence could interact with the WK box(TTTTCCAC) of salicylic acid-inducible defense gene(PR-1a), which stimulated PR-1a gene activity by salicylic acid or bacterial elicitors (van Verk et al., 2008). Furthermore, *Chrysanthemum morifolium* CmWRKY17 with the sequence WKKYGEK could bind to W-box and enhance salinity sensitivity in both chrysanthemum and Arabidopsis (Li et al., 2015). Therefore, the functional implications of WRKYGQK and WRKYGKK variations observed in *GpWRKY* genes warrant further investigation to elucidate their regulatory roles in downstream gene targets and their expression patterns.

Previous studies have reported that *cis*-elements in promoters largely regulate gene expression at the transcriptional level during plant growth and development (Hernandez-Garcia and Finer, 2014). The promoters of *SfWRKY* genes contain hormone-related, stress-related and development-related *cis*-acting elements, indicating *SfWRKY* genes have significant roles in plant growth, biotic and abiotic stress responses (Li et al., 2024). In this study, the hormone-related, stress-related, light-related, and development-related elements were identified in promoters of *GpWRKY* genes, with the light-related and hormone-related elements being more widely distributed, which suggests the *GpWRKY* genes are closely related to plant growth and development (**Figure 5**). The *WRKY* genes play an important role in specific tissues, influencing plant growth and development (Wang et al., 2023a; Yang et al., 2025). *AtWRKY75* had a positive regulatory role in Arabidopsis leaf growth and development (Zhang et al., 2021), while *AtWRKY1* was involved in regulating flowering time and leaf senescence (Zhang et al., 2024a). In addition, *FvWRKY50* was reported to be involved in flowering time, leaf senescence, and fruit ripening (Chen et al., 2023b). Investigating the gene expression patterns of *GpWRKY* in different tissues is essential for *GpWRKY* functional genes. In this study, RNA-seq analysis was performed to investigate the expression patterns of 64 *GpWRKY* genes in the roots, stems, leaves, flowers and fruits of *G. pentaphyllum* (**Figure 7**). Our results revealed that several *GpWRKY* genes were high expressed in specific tissues, including *GpWRKY9* and *GpWRKY55* in roots, *GpWRKY30* and *GpWRKY3*6 in stems, *GpWRKY45* and *GpWRKY51* in leaves, *GpWRKY56* and *GpWRKY13* in flowers, *GpWRKY25* and *GpWRKY52* in fruits, indicating their potentially crucial roles in these organs.

Recent research demonstrates that *G. pentaphyllum* exhibits remarkable adaptability to diverse ecological conditions, thriving across extensive geographical ranges, coupled with significant Cd tolerance and uptake capacity (Li et al., 2023; Nookabkaew et al., 2016). Under Cd exposure, the expression of transcription such as *MYB*, *ERF*, *bZIP*, *WRKY*, *bHLH*, and *GRAS* is modulated, activating pathways involved in phenylpropanoid biosynthesis, starch and sucrose metabolism, and α-linolenic acid metabolism in *G. pentaphyllum* (Zhou et al., 2023). These characteristics support its potential as a species for phytoremediation and for studying mechanisms of Cd contamination response. WRKY transcription factors are known to play an important role in influencing plant responses to metal stresses (Huang et al., 2024b). Overexpression of *PyWRKY75* in poplar significantly enhanced the Cd uptake and accumulation, indicating its role in Cd tolerance (Wu et al., 2022). In contrast, knockdown of the *ZmWRKY64* in maize promoted Cd accumulation in leaf cells and root cytosol, suggesting its importance in coping with Cd stress (Gu et al., 2024). However, although *G. pentaphyllum* is a promising candidate for phytoremediation, the response of *GpWRKY* under Cd stress remains uncharacterized. To explore the role of *GpWRKY* genes under Cd stress, we analyzed the expression of 64 *GpWRKY* genes and identified 13 that were differentially expressed (**Figure 8**). Furthermore, overexpression *GpWRKY48* in Arabidopsis improved growth and elevated the activity of antioxidant enzymes(POD, SOD, CAT), along with a reduction in MDA level under Cd stress, indicating that it functions as a positive regulator of Cd tolerance. These results are consistent with studies of WRKY homologs in other species. Overexpression of *PyWRKY71* and *PyWRKY75* in poplar improved biomass, chlorophyll content, and antioxidant enzyme activities under Cd stress (Chen et al., 2023a; Wu et al., 2022). Similarly, *GmWRKY172-*overexpressing soybeans showed reduced MDA and hydrogen peroxide (H_2_O_2_) accumulation, alongside increased flavonoid and lignin contents, and POD activity under Cd stress (Xian et al., 2023). Overexpression of poplar *PsnWRKY95* significantly enhanced Cd tolerance in transgenic tobacco plants, evidenced by increased plant height, root length, chlorophyll content, and POD activity along with reduced MDA content compared to the wild type (Ma et al., 2025). Together, these findings suggest that the Cd-induced WRKY genes generally act as positive regulators of Cd tolerance. Further functional studies are needed to elucidate the roles of other Cd-responsive *GpWRKY* genes in *G. pentaphyllum*.

## Conclusion

In summary, the *G. pentaphyllum* WRKY gene family was comprehensively analyzed in terms of its structural properties. A total of 64 *GpWRKY* genes were characterized and further classified into seven subfamilies(I, IIa, IIb, IIc, IId, IIe, and III). The physicochemical properties of the GpWRKY proteins varied significantly, while their protein structure and motifs composition exhibited a high degree of conservatism. The 64 *GpWRKY* genes were randomly distributed across 11 chromosomes, with 7 pairs of segmental and tandem duplication events. Expression profiling revealed that the *GpWRKY* genes display tissue-specific expression during growth and development and are responsive to Cd stress. Additionally, *GpWRKY48* was verified to be a positive regulator of Cd tolerance. These findings provide valuable insights into understanding the roles of *GpWRKY* in the growth and stress responses of *G. pentaphyllum*.

## Data Availability

The raw sequence data reported in this paper have been deposited in the Genome Sequence Archive in National Genomics Data Center, China National Center for Bioinformation/Beijing Institute of Genomics, Chinese Academy of Sciences (GSA: CRA031216) that are publicly accessible at https://ngdc.cncb.ac.cn/gsa/browse/CRA031216.
